# Endothelial KDM5B Regulated by Piezo1 Contributes to Disturbed Flow Induced Atherosclerotic Plaque Formation

**DOI:** 10.1111/jcmm.70237

**Published:** 2024-12-06

**Authors:** Lili Wu, Shanshan Jiang, Xiao Zhou, Wei Li, Jiaqi Ke, Ziting Liu, Lijie Ren, Qiongyu Lu, Fengchan Li, Chaojun Tang, Li Zhu

**Affiliations:** ^1^ Cyrus Tang Medical Institute Soochow University Suzhou China; ^2^ Collaborative Innovation Center of Hematology of Jiangsu Province Soochow University Jiangsu Province China; ^3^ Suzhou Key Laboratory of Thrombosis and Vascular Biology Suzhou China; ^4^ National Clinical Research Center for Hematologic Diseases The First Affiliated Hospital of Soochow University Suzhou China; ^5^ Jiangsu Key Laboratory of Preventive and Translational Medicine for Geriatric Diseases Soochow University Suzhou China; ^6^ Suzhou Ninth Hospital affiliated to Soochow University Soochow University Suzhou China

**Keywords:** atherosclerosis, disturbed flow, endothelial cells, KDM5B, Piezo1

## Abstract

Epigenetic modifications play an important role in disturbed flow (d‐flow) induced atherosclerotic plaque formation. By analysing a scRNA‐seq dataset of the left carotid artery (LCA) under d‐flow conditions, we found that Jarid1b (KDM5B) was upregulated primarily in a subcluster of endothelial cells in response to d‐flow stimulation. We therefore investigated the mechanism of KDM5B expression and the role of KDM5B in endothelial cell. Intriguingly, activation of Piezo1, a major endothelial mechanosensor, was found to promote KDM5B expression, which was reversed by Piezo1 inhibition in HUVECs. Downstream of Piezo1, ETS1 expression and c‐JUN phosphorylation were enhanced by d‐flow or Piezo1 activation, leading to an increase in KDM5B expression. Furthermore, knockdown of either KDM5B or Piezo1 was found to prevent d‐flow induced H3K4me3 demethylation, which was supported by the pharmacological inhibition of Piezo1 in HUVECs. RNA sequencing on sh*Kdm5b* HUVECs implied that KDM5B is associated with endothelial inflammation and atherosclerosis. Using partial carotid ligation surgery on *Kdm5b* ^*f/f*^ *Cdh5* ^*cre*^ mice with *mAAV*‐*PCSK9* ^D377Y^ infected, we showed that endothelial KDM5B deficiency reduced atherosclerotic lesions in hypercholesterolemic mice. Our findings indicate that endothelial KDM5B expression induced by d‐flow via the Piezo1 pathway promotes atherosclerotic plaque formation, providing targets for the prevention or therapeutic intervention of atherosclerosis.

## 1 Introduction

Homeostasis in the vascular system plays an important role in the pathogenesis of cardiovascular diseases such as atherosclerosis, stroke and peripheral vascular diseases [1]. The vascular endothelium, which serves as a continuous single cellular lining of the cardiovascular system, represents a central site for critical regulatory junctions within this homeostatic network. By functioning as a monolayer in direct contact with circulating blood, endothelial cells are primarily exposed to wall shear stress and are tasked with responding to pathophysiological stimuli to maintain vessel integrity and homeostasis [2, 3, 4, 5, 6]. Endothelial cells can sense mechanical stimuli and affect cellular function through intracellular signal transduction, such as proliferation, activation, migration and gene expression [7]. Endothelial dysfunction usually occurs in lesion‐prone areas of the arterial vasculature, resulting in increased vascular permeability, cytokine release and leukocyte adhesion [5, 8, 9]. Atherosclerosis occurs preferentially in the aortic arch (AA) and branches of arteries with disturbed flow, and less commonly in areas of laminar blood flow such as the thoracic aorta (TA) [10, 11, 12, 13]. Understanding the effects of d‐flow on endothelial function and differential gene expression will help to define the mechanisms underlying the role of complex flow patterns during plaque formation in atherosclerosis.

Emerging scientific findings underscore the pivotal role of epigenetic alterations in both the initiation and progression of atherosclerosis, as well as in the formation and instability of atherosclerotic plaques [14, 15, 16, 17, 18]. These epigenetic processes involve DNA methylation [19], histone posttranslational modification (PTM) [20, 21] and noncoding RNA [22, 23]. Recent studies on epigenetic modifications driven by fluid dynamics have revealed that flow‐induced shear stress modulates endothelial gene expression through various mechanisms, including DNA methylation [24, 25, 26] and histone acetylation [20, 27]. Histone post‐translational modifications (PTMs) via methylation constitute a crucial and prevalent form of chromatin modification that is widely recognised for its influence on pathobiological processes. For example, laminar blood flow is correlated with a reduction in H3K27 methylation, a chromatin modification that enhances endothelial anti‐inflammatory gene expression [20]. However, how disturbed flow regulates endothelial histone demethylase expression and histone demethylation remains elusive and may aid in understanding the mechanism of atherosclerosis.

KDM5B (also known as JARID1B and PLU1), a histone lysine H3K4me2/me3 demethylase, has been implicated in embryonic development [28], tumorigenesis [29, 30] and treatment resistance [31]. Recent studies have shown that KDM5B can regulate MANTIS, an endothelial cell lncRNA that regulates endothelial cell inflammation and angiogenesis [32]. KDM5B deficiency can promote transcriptional activation of the ATF3 gene transcriptional activation by maintaining H3K4 methylation, reduce myocardial fibrosis, improve cardiac dysfunction [33] and licence macrophage‐mediated inflammatory responses [34]. However, whether disturbed flow controls KDM5B‐mediated histone demethylation and its subsequent potential to modulate endothelial gene expression and contribute to the development of atherosclerosis remain unknown. By analysing a scRNA‐seq dataset of left carotid arteries (LCAs) subjected to disturbed flow (d‐flow) conditions, we found that KDM5B was significantly upregulated in endothelial cells. We thus investigated the mechanisms regulating endothelial KDM5B expression under d‐flow conditions and explored the potential contribution of vascular KDM5B to atherosclerosis progression in mice. We showed that endothelial KDM5B expression induced by d‐flow was regulated by Piezo1 and KDM5B may contributes to atherosclerosis potentially by regulating endothelial cell inflammation.

## 2 Materials and Methods

### 2.1 Animals

All animals described in this study were housed in a specific pathogen‐free facility, and all experiments were approved by the University Committee on Animal Care of Soochow University (20211231). Wild‐type and *ApoE* ^*−/−*^ mice (C57BL/6J background) were purchased from Jackson Laboratories (Bar Harbour, ME, USA). We generated *Kdm5b* conditional knockout mouse (*Kdm5b* ^*f/f*^) with its exon 1 and exon 2 flanked by loxP sites via CRISPR/Cas9 tool on a C57BL6 background. *Kdm5b* ^*f/f*^ *Cdh5* ^*cre*^ mice were generated by crossing *Kdm5b* ^*f/f*^ mice with *Cdh5* ^*Cre*^ mice. At the age of 6–8 weeks, the mice were injected with *mAAV*‐*PCSK9* ^D377Y^ (5 × 10^11^ VG/mouse, Vigene Biosciences, Shandong, China) via tail vein [35, 36]. One week after injection, the mice were subjected to PCL surgery and fed a high‐fat diet (HFD) (0.15% cholesterol and 21% fat without added cholate; Harlan Teklad, 88,137, USA). Before the experiment, mice were anaesthetised by intraperitoneal injection of 1% pentobarbital (7 μL/g). Mice subjected to partial carotid ligation (PCL) surgery were performed under isofurane anaesthesia (3%). The anaesthesia degree of mice was examined by the response of pinching toes. At the end of all experiments, mice were euthanised by CO_2_ inhalation at a rate of 1.9–4.4 L/min for 20 min, followed by cervical dislocation.

### 2.2 Reagents

KDM5‐IN‐1 (Cat# HY‐10042), GsMTx4 (Cat# HY‐P1410), TK216 (HY‐122903), SP600125 (HY‐12041), Yoda1 (Cat# HY‐18723) and Dil (Cat# HY‐D0083) were purchased from MedChemExpress (New Jersey, USA). Recombinant human TNF‐α protein (Cat# HZ‐1014) was purchased from Proteintech (Wuhan, Chian). Fluo‐4 AM dye (Cat# S1060, Beyotime, China) was used to detect calcium flux. Piezo1 polyclonal antibody (Cat#15939‐1‐AP) was purchased from Proteintech (Illinois, USA). An ETS1 monoclonal antibody (Cat# MA5‐15609) was purchased from Invitrogen (California, USA). The primary antibodies used in this study included rabbit anti‐mouse CD31 (1:100, Abcam, Cat# ab28364), rat anti‐mouse CD31 (1:100, BD, Cat# 553700), rabbit anti‐H3K4me3 (Cat# 9751, Cell Signalling Technology, Massachusetts, USA), rabbit anti‐c‐JUN (phospho S63) (Cat# ab32385, Abcam, UK), rabbit anti‐c‐JUN (Cat# ab40766, Abcam, UK), rabbit anti‐KDM5B (Cat# ab181089, Abcam, UK), rabbit anti‐LDLR (SDT‐057‐57, STARTER, China), mouse anti‐VCAM‐1 (Cat# sc‐13160, santa cruz, USA), mouse anti‐ICAM‐1 (Cat# sc‐107, santa cruz, USA) and rabbit anti‐human CD31 (Cat# 28364, Abcam, UK). The secondary antibodies Alexa Fluor 488‐conjugated donkey anti‐rat IgG, Alexa Fluor 488‐conjugated donkey anti‐rabbit IgG, Alexa Fluor 488‐conjugated goat anti‐mouse IgG, Alexa Fluor 647‐conjugated donkey anti‐rabbit IgG, Alexa Fluor 647‐conjugated donkey anti‐mouse IgG, Alexa Fluor 647‐conjugated goat anti‐mouse IgG and Alexa Fluor 568‐conjugated donkey anti‐rabbit IgG were obtained from Abcam. DAPI was purchased from Southern Biotech (Cat# 0100‐01, Alabama, USA) and Beyotime Biotechnology (Cat# C1002, Beyotime, China).

### 2.3 RNA Sequencing Analysis

Genome‐wide gene expression analyses were performed using RNA sequencing (RNA‐seq). Primary sh*Kdm5b* HUVECs were divided into three biological replicates, and total RNA was extracted using an RNA Simple Total RNA Extraction Kit (TIANGEN, Beijing, China, #DP419) according to the manufacturer's instructions. DNBseq sequencing was performed by the BGI Company (Shenzhen, China). Genes with a fold change > 8 and *p* < 0.05 were identified as differentially expressed genes and analysed using online analysis sites.

### 2.4 Lentiviral Infection of HUVECs

Viral vector construction was performed by GeneChem Corporation (Shanghai, China). To knock down *Kdm5b*, HUVECs at 75%–80% confluence were used for transfection. The viral titre of each well was 1 × 10^9^TU and after adding 20 μL of the cotransfection reagent HiTransG, the HUVECs were placed in a 37°C, 5% CO_2_ incubator and the medium was changed to complete medium after 4–6 h. The fluorescence intensity was observed under an inverted fluorescence microscope after 24 h. The sequence of the negative control RNAi (shControl) was TTCTCCGAACGTGTCACGT. The target sequence of *Kdm5b*‐RNAi was CGGCATTCTTTGAGTAGCCTT. shRNA‐mediated KDM5B knockdown in stable cells was tested by real‐time PCR and Western blotting.

### 2.5 Lentiviral Infection of Carotid Arteries

The mouse left carotid artery (LCA) was exposed, and 100 μL of lentiviral suspension (10^7^ TU) was applied to the LCA for 30 min. The skin wound was then closed with silk sutures. After 7 days, the efficiency of target gene knockdown in the infected LCA was examined. Two weeks later, the *ApoE* ^*−/−*^ mice underwent PCL surgery and were reinfected with lentivirus. Lentiviruses for Piezo1 or KDM5B knockdown as well as negative control lentivirus (shControl) were purchased from GeneChem Corporation (Shanghai, China). The target sequence for *Kdm5b* was 5′‐GCCTACATCATGTGAAAGAAT‐3′ and that for *Piezo1* was 5′‐CCTTGTTCCAGGTCTACTA‐3′. The shCtrl sequence was 5′‐TTCTCCGAACGTGTCACGT‐3′.

### 2.6 Disturbed Flow In Vivo by Partial Carotid Ligation

PCL was performed as described previously [24, 37]. In brief, the left carotid artery (LCA) was exposed by blunt dissection. Under a light microscope, three (left external carotid, internal carotid and occipital artery) of the four branches of the LCA were ligated using a 7–0 silk suture. The superior thyroid artery was left untied, providing the sole source for blood circulation. The incision was then closed and cleaned with betadine, and the animals were kept on a heating pad until they regained consciousness. Ultrasound was used to examine blood flow after 24 h following the procedure. When the blood flow in the left carotid artery decreased by approximately 70%–80% compared to that in the right carotid artery, the in vivo PCL model was successfully established.

### 2.7 Analysis of Atherosclerotic Lesions

Atherosclerotic plaques were quantified in *en face* preparations stained with Sudan IV as described previously [38]. Briefly, upon PCL, *ApoE* ^*−/−*^ mice were infected with lentiviral shRNA‐KDM5B or shRNA‐Ctrl in the left common carotid artery. Then, *ApoE* ^*−/−*^ mice were fed a HFD for 2 weeks, or *mAAV*8‐*PCSK9* ^D377Y‐^infected mice were fed a HFD for 3 weeks. Atherosclerotic plaques were stained with Sudan IV. Images of the carotid artery were taken using an Olympus SZX16 telescope (Olympus, Japan) and analysed with Olympus cellSens Standard software (Japan).

### 2.8 Immunofluorescence Staining

Vessels from the TA and carotid artery were fixed in 4% paraformaldehyde overnight and washed with PBS for 5 min three times before dehydrating with 20% sucrose. Tissues were embedded in optimal cutting temperature compound and stored at −80°C. Frozen sections (8 μm) were incubated with primary antibodies or an IgG control at 4°C overnight, followed by incubation with fluorescent secondary antibodies for 1 h and DAPI for 15 min. Images were obtained using a multicolor digital camera on an FV3000 confocal microscope (Olympus, Japan).

### 2.9 Primary Human Umbilical Vein Endothelial Cell Culture

Primary human umbilical vein endothelial cells (HUVECs) (Cat#PCS‐100‐010, ATCC, Maryland, USA) were maintained in vascular cell basal medium (Cat#PCS‐100‐030, ATCC, Maryland, USA) supplemented with ascorbic acid (Cat#PCS‐999‐006), FBS (Cat#PCS‐999‐010), rhEGF (Cat#PCS‐999‐018), heparin sulfate (Cat#PCS‐999‐011), L‐glutamine (Cat#PCS‐999‐01), rhVEGF (Cat#PCS‐999‐02), rhFGF‐b (Cat#PCS‐999‐020), rhIGF‐1 (Cat#PCS‐999‐021) and hydrocortisone (Cat#PCS‐999‐014, ATCC, Maryland, USA).

### 2.10 Preparation of Cell Lysates and Immunoblotting

Cells were lysed on ice using RIPA lysis buffer (1% Triton X‐100, 1% deoxycholate, 0.1% SDS, 10 mM Tris and 150 mM NaCl) containing protease and phosphatase inhibitor cocktail (Santa Cruz Biotechnology Inc. Heidelberg, Germany). The cell lysates were sonicated by ultrasonication (Sonics, China, Cat# VCX130) and centrifuged at 14,000 × *g* for 5 min at 4°C. The protein concentration of the supernatant was measured using a BCA protein assay kit (Beyotime, China). The reduced proteins (30 μg) in 4 × sample buffer (Invitrogen) containing β‐2‐mercaptoethanol were heated at 95°C for 5 min before loading. Protein samples were separated on a 10% gel and transferred to nitrocellulose filter membranes. The membranes were blocked with 5% nonfat dry milk (Bio‐Rad, California, USA) in TBS‐T and incubated with primary antibody overnight at 4°C, followed by incubation with fluorescent secondary antibodies for 1 h at room temperature. After washing, the membranes were scanned using an Odyssey infrared imaging system (LI‐COR Biosciences, USA). Densitometric analysis was performed using ImageJ software (NIH) to quantify protein expression levels, with β‐actin serving as an internal control.

### 2.11 HEK 293 Cell Culture and Luciferase Reporter Assay

The human embryonic kidney cell line 293T (HEK 293T) was obtained from the cell bank of the Chinese Academy of Sciences (Shanghai, China). HEK293T cells were cultured in Dulbecco's modified Eagle's medium supplemented with 10% fetal bovine serum (HyClone, Utah, USA). The cell cultures were maintained at 37°C in a humidified 5% CO_2_ incubator (Thermo Forma Electron Co., Ohio, USA).

The luciferase reporter vector GV238‐basic vector with a −2000/−1 bp region of the KDM5B gene was constructed. After digestion by Xhol and Kpnl, the fragments of the −2000/−1 bp region of the KDM5B gene were cloned and inserted into the Xhol and Kpnl sites of the reporter luciferase vector and named KDM5B pro‐Luc. HEK293T cells were seeded in 96‐well plates 12 h prior to transfection. In each well, cells were transfected with 0.1 μg KDM5B pro‐Luc plasmid or empty luciferase reporter plasmid (empty‐Luc) as a control, together with 0.1 μg GV230‐basic vector containing ETS1 plasmid (ETS1‐GV230) or c‐JUN plasmid (c‐JUN‐GV230) with empty vector (empty‐GV230) as a control. Transfection was performed using Lipofectamine 3000 (Cat# L3000150; Invitrogen, California, USA). Assays were carried out with a dual luciferase reporter assay system (Cat# E1500, Promega, Wisconsin, USA).

### 2.12 Shear Stress Stimulation of HUVECs In Vitro

A modified cone‐and‐plate shear stress device was used to generate oscillatory shear stress (OS) and laminar shear stress (LS) in vitro [17]. HUVECs cultured in a 10 cm petri dish (70% confluent) were stimulated with OS (10 ± 5 dyn/cm^2^) for 24 h or with LS (30 dyn/cm^2^) for 24 h as a control. The culture plates and the shear stress device were kept in an incubator with 5% CO_2_ at 37°C.

### 2.13 Real‐Time Quantitative PCR (RT‐qPCR)

Total RNA from the LCA or RCA was extracted using the QIAGEN miRNeasy Mini kit (217,004, Qiagen, Germany). Total RNA was reverse transcribed into cDNA using PrimeScript RT Master Mix (TAKARA, Japan). qPCR was performed in triplicate in 20 μL of the Brilliant SYBR Green PCR Master Mix (4,913,914, Roche, Switzerland) in a real‐time PCR system (LightCycler 480, Roche, Switzerland). The mRNA expression levels were normalised to the glyceraldehyde‐3‐phosphate dehydrogenase (GAPDH) level and are presented as relative fold changes determined by the 2‐ΔΔCT method [39]. The sequences of primers used for each gene are given in the Supporting Information, Table S1.

### 2.14 Online Database Analysis

The conserved transcription factor binding sites (TFBS) of ETS1 (MA0098.2) and c‐JUN (MA0488.1) were indicated based on the JASPAR database (Sandelin, et al., 2004).

The results of the sequencing of the KDM5B gene were analysed using the BLAT Tool (UCSC Genome Browser, https://genome‐euro.ucsc.edu/). The putative transcription factors of KDM5B within a dissimilarity margin less than or equal to 5% were predicted by using the online bioinformatics tool PROMO alggen.

### 2.15 Statistics

Statistical analysis was performed with GraphPad Prism 8.0 (GraphPad Software, San Diego, CA, USA). All the data are expressed as the mean ± standard error of the mean (mean ± SEM), and *p* < 0.05 was considered to indicate statistical significance. Comparisons of data from different experimental groups were conducted using unpaired Student's t‐tests (two‐tailed) and two‐way ANOVA tests with post hoc analysis. Violin plot analysis was performed using the Fluidigm Singular Analysis ToolSet 3.5.2R package.

## 3 Results

### 3.1 The Histone Demethylase KDM5B Is Expressed in Vascular Endothelial Cells Under Disturbed Flow Conditions

**FIGURE 1:**
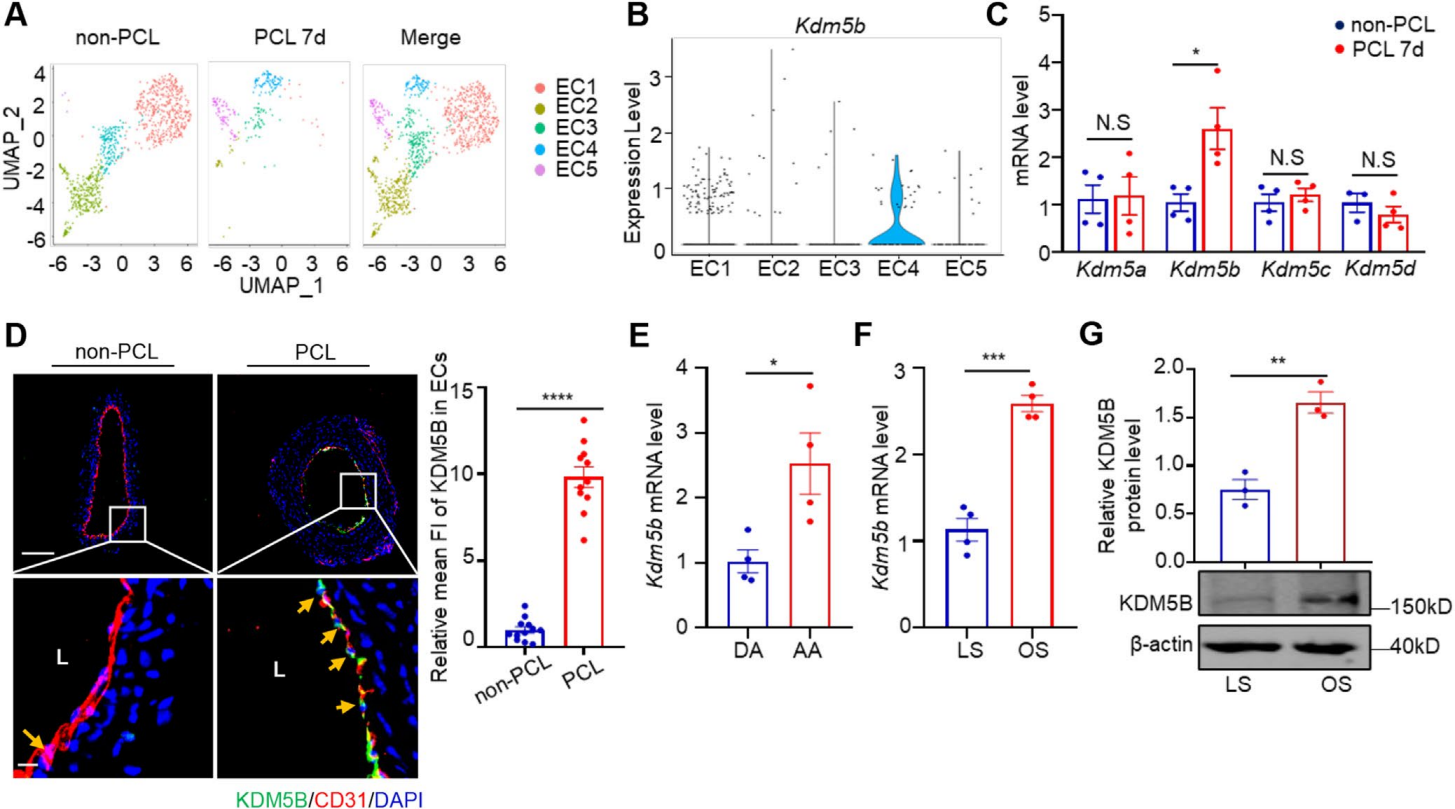
The histone demethylase KDM5B is expressed in vascular endothelial cells under disturbed flow conditions. (A) UMAP plot illustrating endothelial cell clusters identified in the left carotid artery of wild‐type mice after PCL 7d or without PCL (non‐PCL). (B) Violin plots of *Kdm5b* expression in five EC clusters. (C) *Kdm5a*, *Kdm5b*, *Kdm5c*, and *Kdm5d* mRNA levels in the carotid artery after PCL 7d or non‐PCL were normalised to those of *Gapdh* and are displayed as fold changes relative to baseline (non‐PCL) (*n* = 4 per group). *p < 0.05 by two‐way ANOVA with the Sidak test for multiple comparisons. (D) KDM5B protein level in carotid endothelial cells of *WT* mice after PCL 7d was measured by IF staining with anti‐KDM5B and anti‐CD31 antibodies, and the results are shown as the fold change compared with those in non‐PCL mice (red, CD31; green, KDM5B; blue, DAPI). The right panel is the enlarged image of the boxed area in the left panel. Left bar = 100 μm, right bar = 20 μm. (*n* = 11 mice per group). ****p < 0.0001 by unpaired Student's *t*‐test. (E) *Kdm5b* mRNA in the aortic arch (AA) and descending aorta (DA) of C57BL/6J mice was assessed by RT‐qPCR, using *Gapdh* as a control (*n* = 4 per group). *p < 0.05 by unpaired Student's *t*‐test. (F) Disturbed flow increases KDM5B expression. *Kdm5b* mRNA expression in HUVECs subjected to laminar shear stress (LS) or oscillatory shear stress (OS) for 24 h was assessed by RT‐qPCR, and *Gapdh* was used as a control (*n* = 4 per group). ***p < 0.001 by unpaired Student's *t*‐test. (G) Western blotting was used to determine the expression of KDM5B (*n* = 3 per group). **p < 0.01 by unpaired Student's *t*‐test.

Histone PTM by methylation is a reversible posttranslational process written by histone methylases and erased by histone demethylases [14]. Although our understanding of histone methylation in vascular biology has improved [40, 41], the role of histone methylases and demethylases in atherogenesis has not been extensively investigated. To profile the endothelial expression of histone demethylases that respond to proatherosclerotic d‐flow stimulation, we analysed a dataset from single‐cell RNA sequencing (scRNA‐seq) of a PCL mouse model [42], which revealed a heterogeneity of vascular endothelial cells, with subclusters EC1, EC2 and EC3 mainly in the non‐PCL carotid artery and emerging subclusters EC4 and EC5 in the PCL surgery group (Figure 1A). Intriguingly, the expression of KDM5B, a member of the histone lysine (K) demethylase KDM5 family (KDM5A‐D, also known as JARID1A‐D), was selectively upregulated in the d‐flow‐stimulated EC4 cluster but not in the other EC clusters (Figures 1B, S1), which was confirmed by *Kdm5b* mRNA expression (Figure 1C) and immunostaining of KDM5B (Figure 1D). Consistently, the mRNA level of *Kdm5b* was upregulated in the aortic arch (AA), a region where d‐flow and atherosclerotic plaques preferentially develop, compared to the descending aorta (DA) (Figure 1E). To directly evaluate the effect of d‐flow on KDM5B expression, using an in vitro cone‐plate device [12], we analysed the expression of KDM5B in HUVECs after stimulation with LS or OS for 24 h. KDM5B mRNA and protein levels in HUVECs exposed to OS were significantly higher than those in cells exposed to LS (Figure 1F,G).

### 3.2 Piezo1 Promotes Endothelial KDM5B Expression Under Disturbed Flow Conditions

**FIGURE 2:**
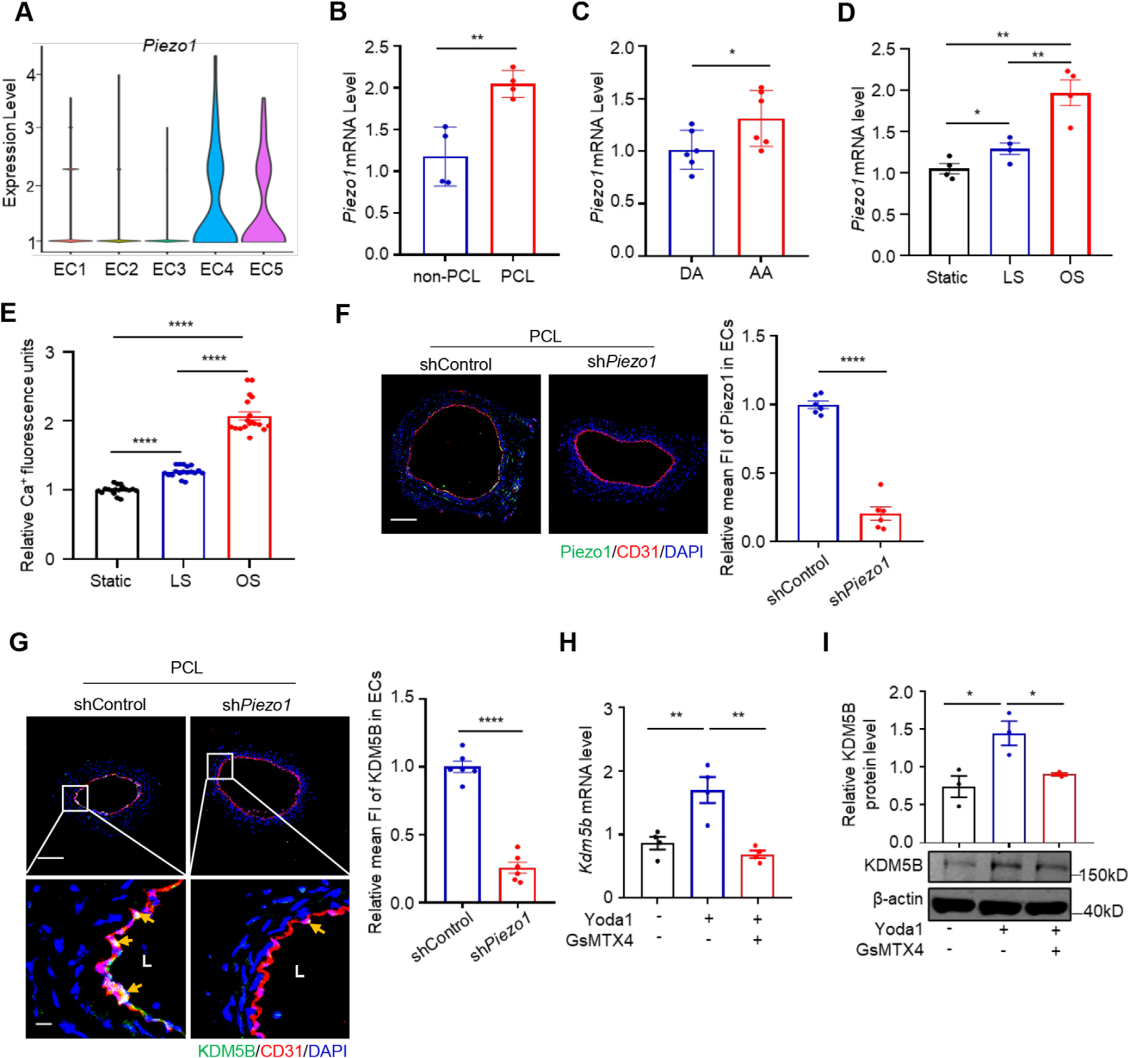
Piezo1 promotes endothelial KDM5B expression under disturbed flow conditions. (A) Violin plots of Piezo1 expression in five EC clusters identified from the left carotid artery of wild‐type mice after PCL 7d or non‐PCL. (B) *Piezo1* mRNA expression in the carotid artery after PCL 7d or non‐PCL surgery was normalised to that of *Gapdh* and is displayed as the fold change relative to baseline (non‐PCL) (*n* = 4 per group). **p < 0.01 by unpaired Student's *t*‐test. (C) *Piezo1* mRNA expression in the aortic arch (AA) and descending aorta (DA) of C57BL/6J mice was assessed by RT‐qPCR, and *Gapdh* was used as a control (*n* = 6 per group). *p < 0.05 by unpaired Student's *t*‐test. (D) Disturbed flow increases Piezo1 expression. *Piezo1* mRNA expression in HUVECs subjected to laminar shear stress (LS) or oscillatory shear stress (OS) for 24 h was assessed by RT‐qPCR, and *Gapdh* was used as a control (*n* = 4 per group). *p < 0.05, **p < 0.01 by unpaired Student's *t*‐test. (E) Shear stress stimulation promotes Piezo1 activation. Fluo‐4 AM prob. were use to detect the concentration of endothelial calcium. ****p < 0.0001 by unpaired Student's *t*‐test. (F, G) Mouse carotid arteries were infected with lentivirus‐mediated shRNA targeting *Piezo1*, and immunostaining was used to determine Piezo1 (F) and KDM5B (G) expression in endothelial cells. Scale bar = 100 μm (F). The right panel is the enlarged image of the boxed area in the left panel, left bar = 100 μm, right bar = 20 μm (G). (*n* = 6 per group). ****p < 0.0001 by unpaired Student's *t*‐test. (H & I) HUVECs were treated with the Piezo1 agonist Yoda1 (10 μM) or the blocking peptide GsMTx4 (500 nM) for 24 h. RT‐qPCR (H) (*n* = 4 per group) and Western blotting (I) (*n* = 3 per group) were used to determine the expression of KDM5B. *p < 0.05 by unpaired Student's *t*‐test.

To investigate how d‐flow induces endothelial KDM5B expression, we screened mechanosensors that are differentially expressed in sh*Kdm5b* HUVECs and analysed their expression in the scRNA‐seq dataset [42] from the LCA after PCL (Figure S2A). We showed that the well‐known mechanosensor Piezo1 was mainly expressed in the d‐flow‐associated EC4 and EC5 subclusters (Figure 2A). Similarly, *Piezo1* mRNA was upregulated in the LCA after PCL and in the aortic arch compared to that in the flow‐protective region (Figure 2B,C). Piezo1 has been reported to be involved in shear stress induced endothelial calcium influx [43, 44, 45]. To test whether disturbed flow contributes to the upregulation and activation of Piezo1, we used an in vitro device and a Fluo‐4 AM prob. and showed that *Piezo1* mRNA expression and Ca^2+^ influx were upregulated in both laminar flow stimulated and disturbed flow stimulated HUVECs compared with static HUVECs, which was consistent with the findings of a previous study [44] (Figure 2D,E). We therefore examined whether Piezo1 is associated with d‐flow induced KDM5B expression by both genetic and pharmacological approaches. We knocked down the mouse *Piezo1* gene in the carotid artery by delivering a lentivirus carrying mouse *Piezo1* shRNA (sh*Piezo1*) to the left common carotid artery. Two days after PCL, we examined the association of Piezo1 with KDM5B expression and showed that *Piezo1* knockdown reduced endothelial KDM5B expression in the carotid artery (Figure 2F,G), indicating that Piezo1 plays a role in mediating d‐flow induced endothelial KDM5B expression. Alternatively, the association of Piezo1 with d‐flow induced KDM5B expression was examined using the Piezo1 agonist Yoda1 and the blocking peptide GsMTx4. Similarly, we found a significant increase in the Ca^2+^ concentration in endothelial cells after agonist stimulation, which was reversed by the addition of blocking peptide (Figure S2B,C) [44, 46]. Besides, treatment of HUVECs with Yoda1 increased the expression of KDM5B, which was inhibited by GsMTx4 treatment (Figure 2H,I), further confirming the role of Piezo1 in regulating endothelial KDM5B expression under disturbed flow conditions.

### 3.3 Piezo1 Upregulates KDM5B Expression via ETS1 and c‐JUN

**FIGURE 3:**
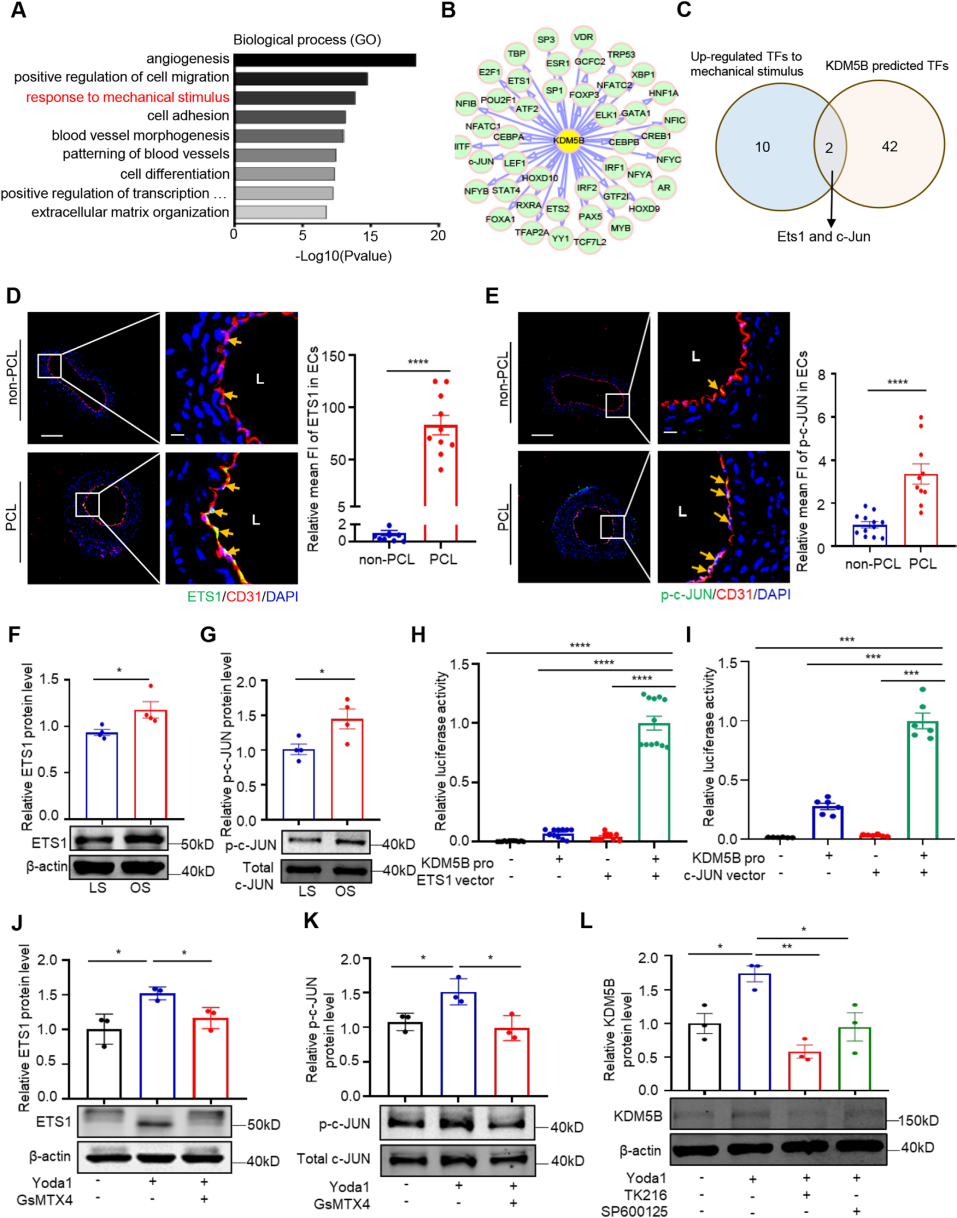
Piezo1 upregulates KDM5B expression via ETS1 and c‐JUN. (A) GO term enrichment analysis in the biological process category of upregulated genes in the PCL‐treated mouse carotid artery according to the scRNA‐seq data. (B) KDM5B predicted transcription factors using the UCSC website and the online bioinformatics tool PROMO database. (C) Venn diagram analysis of overlapping genes among the genes upregulated in response to mechanical stimulus and among the predicted KDM5B TFs. (D, E) Immunofluorescence staining of ETS1/c‐JUN (green), CD31 (red), and DAPI (blue); ETS1 expression (D) and c‐JUN expression (E) were decreased in carotid endothelial cells after PCL7d treatment compared with those after non‐PCL treatment. The right panel is the enlarged image of the boxed area in the left panel. Left bar = 100 μm, right bar = 20 μm. (*n* ≥ 10 mice per group), ****p < 0.0001 by unpaired Student's *t*‐test. (F & G) HUVECs were treated for 24 h with OS or LS before Western blotting to examine the expression of ETS1 (F) or c‐JUN (G). (*n* = 3 per group), *p < 0.05 by unpaired Student's *t*‐test. (H, I) The KDM5B luciferase reporter was cotransfected with the ETS1 vector (H) or c‐JUN vector (I) into HEK293T cells, and luciferase activity was evaluated 24 h later. The data are presented as the mean ± SEM. (*n* ≥ 6 per group), ***p < 0.001, ****p < 0.0001 by unpaired Student's *t*‐test. (J, K) Immunoblotting results showing the protein levels of ETS1 (J) and c‐JUN (K) in HUVECs treated with vehicle, Yoda1 (10 μM) or GsMTx4 (500 nM) for 24 h. *p < 0.05 by unpaired Student's *t*‐test. (L) Western blot analysis of extracts from HUVECs treated with Yoda1 (10 μM) for 24 h and then treated with TK216 (2 μM, 4 h) or SP600125 (10 μM, 40 min). Data are presented as the mean ± SEM. (*n* = 3 per group), *p < 0.05, **p < 0.01 by unpaired Student's *t*‐test.

We next investigated how Piezo1 regulates d‐flow induced KDM5B expression by examining genes that mediate the effect of Piezo1 on KDM5B expression. We first screened the key genes that respond to mechanical stimuli in endothelial cells of the carotid artery after 7 days of PCL surgery by scRNA‐seq analysis. GO analysis revealed changes in 12 genes enriched in ‘response to mechanical stimulus’ (Figures 3A, S3). We then predicted KDM5B transcription factors using the UCSC website and the online bioinformatics tool PROMO alggen database [47, 48]. Strikingly, ETS1 and c‐JUN overlapped with genes responsive to mechanical stimuli in 44 predicted KDM5B transcription factors (Figure 3B,C). Moreover, immunostaining revealed higher levels of endothelial ETS1 and phospho‐c‐JUN in LCA with PCL surgery than that in non‐PCL RCA (Figure 3D,E). Consistently, the levels of ETS1 and phospho‐c‐JUN in the HUVECs exposed to OS were significantly higher than those in the cells exposed to LS (Figure 3F,G). These results suggested the involvement of endothelial ETS1 and phospho‐c‐JUN in endothelial KDM5B expression under disturbed flow conditions.

Piezo1‐induced Ca^2+^ influx [39] and blood flow stimulation [49] can promote the phosphorylation of ETS1, thereby regulating gene transcription and expression. To determine whether *Kdm5b* is the target gene of ETS1 and c‐JUN, a luciferase reporter assay was used to examine the binding of ETS1 and c‐JUN to the *Kdm5b* promoter region in HEK293T cells (Figure S4). The results showed that the luciferase activity of the reporter gene containing the *Kdm5b* promoter region was greater after transfection with the ETS1 or c‐JUN plasmid than that after transfection with the vector control (Figure 3H,I), indicating that ETS1 and c‐JUN bind to the *Kdm5b* promoter region. We then determined the effect of Piezo1 signalling on ETS1 expression and c‐JUN activation in endothelial cells. The results showed that Piezo1 activation by Yoda1 resulted in ETS1 upregulation and c‐JUN phosphorylation, which was reversed when Piezo1 was inhibited by GsMTx4 (Figure 3J,K). Using the ETS1 inhibitor TK216 and the c‐JUN inhibitor SP600125, we showed that the inhibition of EST1 or c‐JUN reduced KDM5B expression by Yoda1 (Figure 3L). Taken together, our data indicated that Piezo1 activation induces the binding of ETS1 and c‐JUN to the *Kdm5b* promoter region, enhancing KDM5B expression in disturbed flow‐stimulated endothelial cells.

### 3.4 The Piezo1‐KDM5B Axis Contributes to Disturbed Flow Induced Demethylation of Endothelial Histone H3K4me3

**FIGURE 4:**
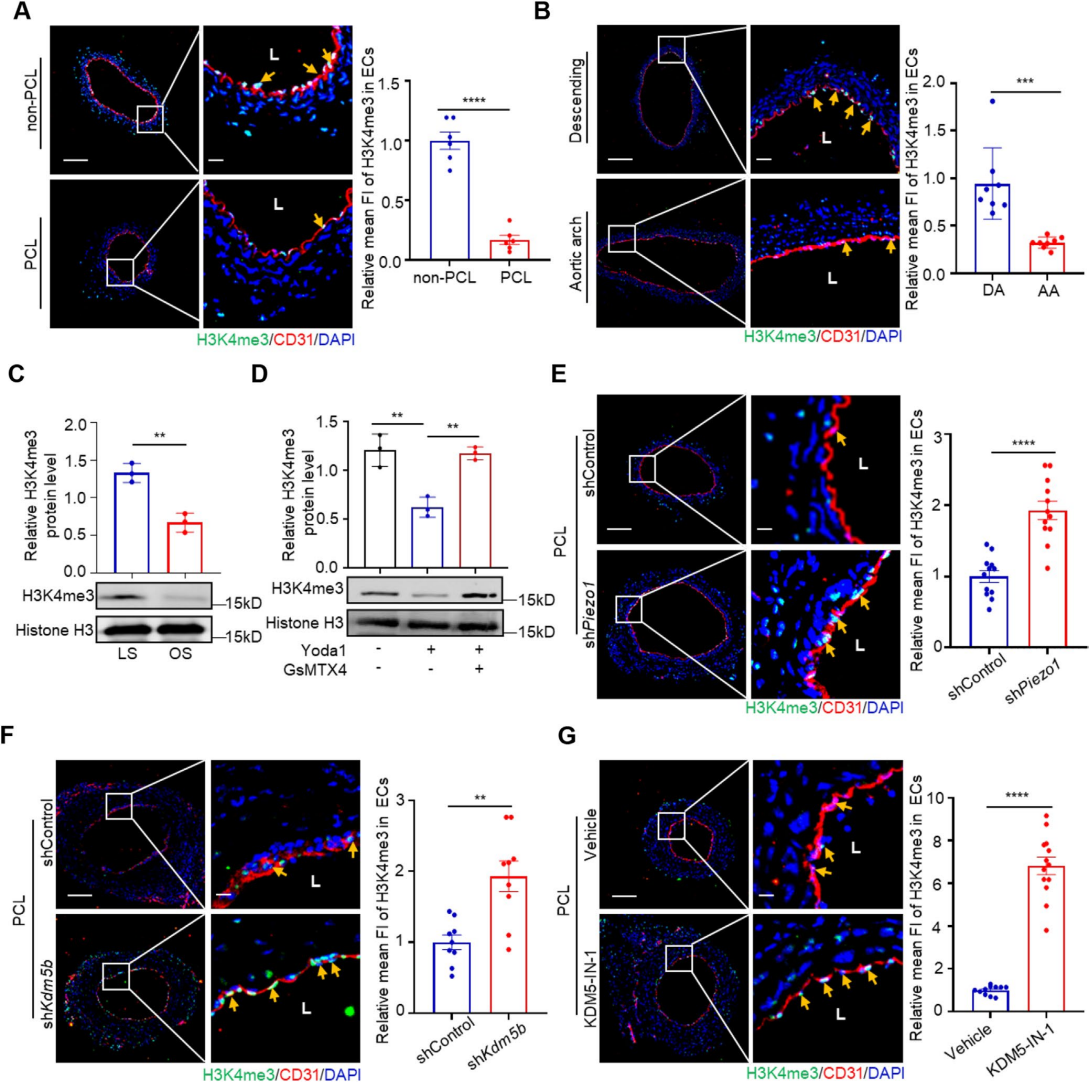
The Piezo1‐KDM5B axis contributes to disturbed flow induced demethylation of endothelial histone H3K4me3. (A) The left carotid arteries (LCA) of mice exposed to disturbed flow were stained for H3K4me3 (green) and CD31 (red) and DAPI (blue) after PCL 7d, and the right carotid arteries (RCA) were used as controls (non‐PCL). Quantitative statistical analysis of H3K4me3 fluorescence intensity in endothelial cells is shown on the right (fold change compared with that in non‐PCL group). The right panel is the enlarged image of the boxed area in the left panel. Left bar = 100 μm, right bar = 20 μm. (*n* ≥ 6 per group). ****p < 0.0001 by unpaired Student's *t*‐test. (B) Immunofluorescence staining for H3K4me3 (green), CD31 (red) and DAPI (blue) in the AA and DA of *WT* mice. Quantification of H3K4me3 fluorescence intensity in endothelial cells is shown on the right (fold change compared with that in the DA group). The right panel is the enlarged image of the boxed area in the left panel. Left bar = 100 μm, right bar = 20 μm. (*n* = 8 per group). ***p < 0.001 by unpaired Student's *t*‐test. (C) HUVECs were exposed to LS or OS for 24 h before Western blotting to examine the expression of the H3K4me3 mark. *p < 0.05 by unpaired Student's *t*‐test. (D) HUVECs were treated with the Piezo1 agonist Yoda1 (10 μM) or the blocking peptide GsMTx4 (500 nM) for 24 h before Western blotting to examine the expression level of the H3K4me3 mark. **p < 0.01 by unpaired Student's *t*‐test. (E) Immunofluorescence staining of H3K4me3 (green), CD31 (red) and DAPI (blue) in mouse carotid arteries infected with lentivirus targeting Piezo1 after PCL 7 days. The right panel is the enlarged image of the boxed area in the left panel. Left bar = 100 μm, right bar = 20 μm. (*n* = 12 mice per group). ****p < 0.0001 by unpaired Student's *t*‐test. (F) Immunofluorescence staining of H3K4me3 (green), CD31 (red) and DAPI (blue) in the mouse carotid artery after instillation of lentivirus targeting KDM5B after PCL for 7 days. The right panel is the enlarged image of the boxed area in the left panel. Left bar = 100 μm, right bar = 20 μm. (*n* = 9 mice per group). **p < 0.01 by unpaired Student's *t*‐test. (G) Representative immunofluorescence staining of H3K4me3 (green) in the carotid artery of mice treated with KDM5‐IN‐1 implantation for 7 days after PCL. The right panel is the enlarged image of the boxed area in the left panel. Left bar = 100 μm, right bar = 20 μm. (*n* ≥ 11 mice per group). ****p < 0.0001 by unpaired Student's *t*‐test.

As KDM5 subfamily members function as H3K4 demethylases that preferentially demethylase trimethyl H3K4 [34, 50], we examined H3K4me3 modification in the carotid arteries after 7 days of PCL and showed a reduced level of H3K4me3 in the endothelial cells under disturbed flow (Figure 4A). Remarkably, the level of H3K4me3 in endothelial cells of the aortic arch was significantly lower than that in the descending aorta (Figure 4B). Consistently, the level of H3K4me3 in HUVECs exposed to oscillatory shear stress (OS) was significantly lower than that in cells exposed to laminar shear stress (LS) (Figure 4C). These results indicated that disturbed flow induces vascular endothelial demethylation of H3K4me3.

We next examined the role of KDM5B and Piezo1 in disturbed flow induced demethylation of endothelial histone H3K4me3. We first found that pharmacological inhibition of Piezo1 by GsMTx4 reduced H3K4me3 demethylation induced by the Piezo1 activator Yoda1 (Figure 4D). Similarly, the effect of Piezo1 knockdown on H3K4me3 demethylation by disturbed flow was evaluated, and the results showed that Piezo1 knockdown protected against disturbed flow induced H3K4me3 demethylation in the mouse carotid artery (Figure 4E). We then used a *Kdm5b*‐based lentiviral vector (sh*Kdm5b*) to knock down the KDM5B expression in the mouse carotid artery to study the regulatory effect of KDM5B on H3K4me3 level under disturbed flow conditions. KDM5B knockdown protected against disturbed flow induced H3K4me3 demethylation in the mouse carotid artery compared with vector shRNA‐transfected mouse carotid artery (Figure 4F). Moreover, we delivered KDM5‐IN‐1, a specific inhibitor of KDM5B and KDM5C, to PCL‐treated mice by oral gavage and found that KDM5‐IN‐1 protected against disturbed flow induced H3K4me3 demethylation in endothelial cells (Figure 4G). Together, these data indicate that the mechanosensor Piezo1 regulates KDM5B expression and Piezo1‐KDM5B axis contributes to disturbed flow induced demethylation of endothelial histone H3K4me3.

### 3.5 KDM5B Knockdown Inhibits Endothelial Inflammation and Reduces d‐Flow Induced Carotid Atherosclerotic Plaque Size in Mice

**FIGURE 5:**
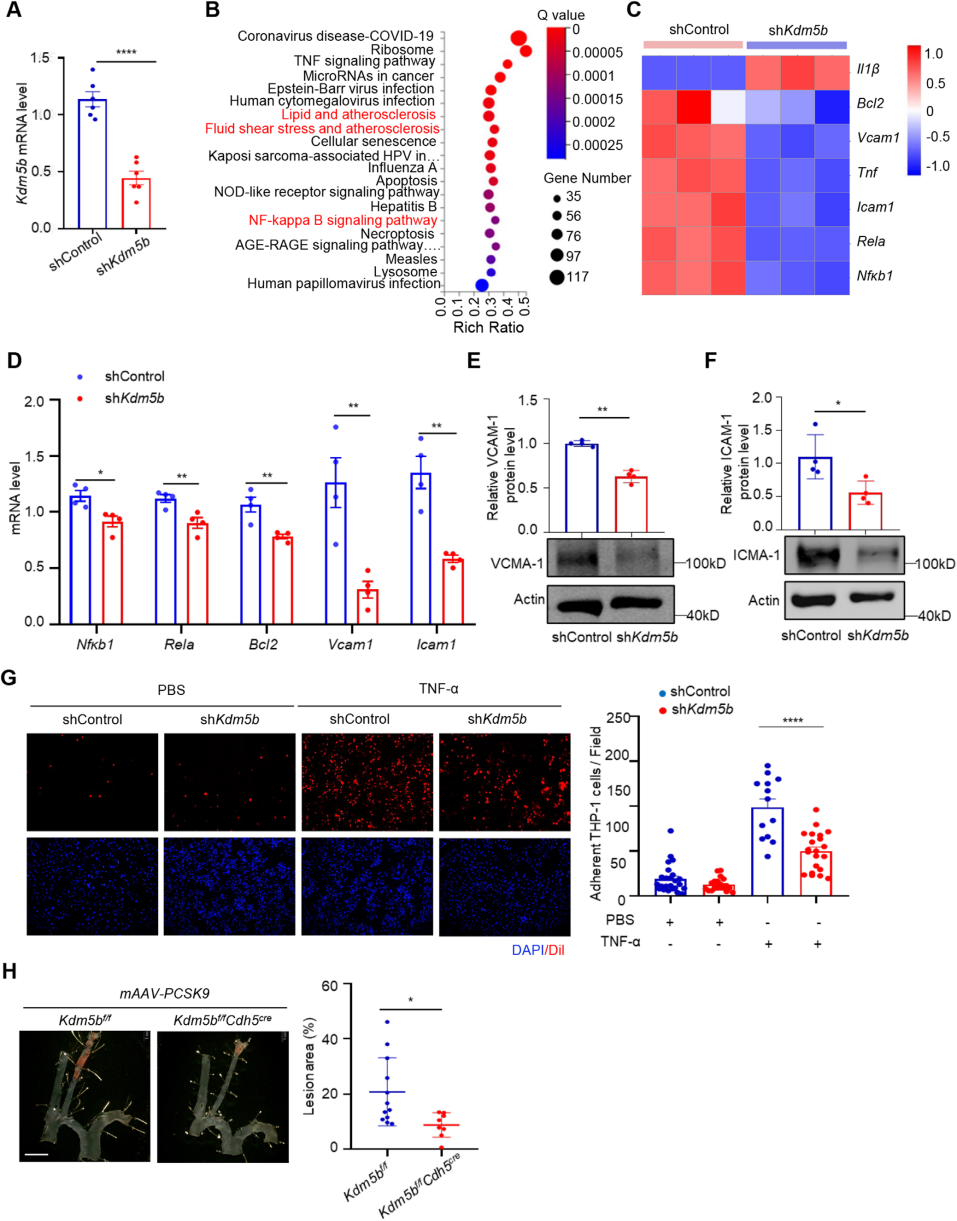
KDM5B knockdown inhibits endothelial inflammation and reduces d‐flow induced carotid atherosclerotic plaque size in mice. (A) *Kdm5b* mRNA expression in lentivirus‐mediated shControl and sh*Kdm5b* HUVECs was analysed by RT‐qPCR (*n* ≥ 5 per group). The data are presented as the mean ± SEM. ****p < 0.0001 by unpaired Student's *t*‐test. (B) Bubble chart showing the signalling pathways enriched with differentially expressed genes in the RNA‐Seq dataset of KDM5B‐knockdown HUVECs according to KEGG analysis. (C) Heatmap showing the genes involved in red marked signalling pathways. (D) HUVECs were transfected with control shRNA or *Kdm5b* shRNA lentivirus. The expression of *Nfkb1*, *Rela*, *Bcl2*, *Vcam1*, and *Icam1* were determined by RT‐qPCR. (*n* = 6 per group). *p < 0.05, **p < 0.01 by unpaired Student's *t*‐test. (E, F) The expression of VCAM‐1 and ICAM‐1 were determined by Western Blot. (*n* = 4 per group). *p < 0.05, **p < 0.01 by unpaired Student's *t*‐test. (G) THP‐1 cells were stained by Dil and incubated with shControl HUVECs or sh*Kdm5b* HUVECs for 30 min at 37°C. Adherent THP‐1 cells (red) on the monolayer of HUVECs were visualised under a microscope and quantified. ****p < 0.0001 by unpaired Student's *t*‐test. (H) *Kdm5b* ^*f/f*^ mice (*n* = 12) and *Kdm5b* ^*f/f*^ *Cdh5* ^*cre*^ mice (*n* = 8) were injected with *mAAV‐PCSK9* (5 × 10^11^ VG/mouse). After 1 week, the mice were subjected to PCL and fed a HFD for 3 weeks. Lipid deposition (in red) in the mouse LCA was analysed by *en face* Sudan IV staining. The lesion surface area was quantified and is displayed as the percentage area of the LCA. Bar = 1 mm. The data are presented as the mean ± SEM. *p < 0.05 by unpaired Student's *t*‐test.

To examine the role of KDM5B in endothelial cells, we generated *Kdm5b*‐knockdown HUVECs by transducing lentiviral vectors expressing human sh*Kdm5b*, and the results were confirmed by quantitative PCR and immunoblotting (Figures 5A, S5A). We then performed RNA sequencing on sh*Kdm5b* HUVECs and shControl HUVECs (Figure S5B). KEGG signalling pathway analysis of the differentially expressed genes revealed enrichment of the ‘lipid and atherosclerosis’, ‘fluid sehear stress and atherosclerosis’, and ‘NF‐κB signaling pathway’ (Figure 5B), suggesting the involvement of endothelial KDM5B in the development of atherosclerosis. Venn analysis of the genes involved in these signalling pathways showed that KDM5B knockdown reduced pro‐inflammatory gene expression, such as VCAM‐1 and ICAM‐1, which were confirmed by RT‐qPCR and Western blotting (Figures 5C–F, S5C). To determine whether KDM5B is involved in leukocyte‐endothelial adhesion, we used THP‐1 cells (a human acute monocytic leukaemia cell line) and HUVECs to perform an adhesion assay. Compared with control HUVECs, KDM5B‐knockdown HUVECs presented a 1.97‐fold decrease in THP‐1 adhesion under TNF‐α stimulation (Figure 5G). These results indicated that KDM5B may affect endothelial inflammation, suggesting an involvement of endothelial KDM5B in the development of atherosclerosis.

To explore the potential role of KDM5B in carotid atherosclerotic plaque formation by disturbed flow, we first knocked down the *Kdm5b* gene in the carotid artery of *ApoE* ^*−/−*^ mice by delivering a lentivirus carrying mouse *Kdm5b* shRNA to the left common carotid artery (Figure S5D). Immunostaining and RT‐qPCR were used to detect KDM5B expression in the carotid artery, and KDM5B was less expressed in sh*Kdm5b*‐infected arteries compared to shControl‐infected arteries (Figure S5E,F). The mice then underwent PCL surgery followed by HFD feeding for 2 weeks. Results showed that the size of atherosclerotic lesions was reduced by 41.45% (*p* < 0.001) in lentiviral *Kdm5b* shRNA‐transfected *ApoE* ^*−/−*^ mice (24.69 ± 1.6%, plaque area in total area, *n* ≥ 7) compared to that in shControl‐transfected *ApoE* ^*−/−*^ mice (42.17 ± 1.9%, plaque area in total area, *n* ≥ 7) (Figure S5G), indicating that *Kdm5b* knockdown attenuates atherosclerotic plaque formation by d‐flow. To further explore the role of endothelial KDM5B in atherosclerotic plaque formation, we generated hypercholesterolemic mice by infecting *Kdm5b* ^*f/f*^ *Cdh5* ^*cre*^ mice and *Kdm5b* ^*f/f*^ littermates with *mAAV*‐*PCSK9* ^D377Y^. After 7 days, immunoblotting showed that LDLR protein levels were largely decreased in mice infected with *mAAV*‐*PCSK9* ^D377Y^ (Figure S5I). Mice then received PCL surgery followed by HFD feeding for 3 weeks (Figure S5H). Atherosclerotic lesion size was reduced by 57.76% (*p* < 0.001) in *Kdm5b* ^*f/f*^ *Cdh5* ^*cre*^ mice (8.789 ± 3.559%, plaque area in total area, *n* ≥ 12) compared to that in *Kdm5b* ^*f/f*^ mice (20.79 ± 1.572%, plaque area in total area, *n* ≥ 8) (Figure 5H), demonstrating that endothelial KDM5B contributes to atherosclerotic plaque formation. As KDM5B was reported to regulate lipid metabolism [51, 52], to determine whether lipid metabolism accounts for the decrease in atherosclerotic plaque development caused by KDM5B deficiency, serum lipid levels were measured after a 12 h fasting period. No overt differences were observed in the serum levels of total cholesterol (TC), triglycerides (TG), high‐density lipoprotein (HDL) or low‐density lipoprotein (LDL) between *Kdm5b* ^*f/f*^ *Cdh5* ^*cre*^ mice and *Kdm5b* ^*f/f*^ controls (Figure S5J–M). Additionally, endothelial KDM5B deficiency did not affect body weight during the development of atherosclerosis (Figure S5N).

## 4 Discussion

In this study, we examined the endothelial expression of the demethylase KDM5B upon flow disturbance and its role in the development of atherosclerosis. We showed that KDM5B was expressed primarily in a subcluster of endothelial cells that respond to disturbed flow. KDM5B knockdown or endothelial deletion attenuated atherosclerotic plaque formation. The mechanosensor Piezo1 and the downstream transcription factors c‐JUN and ETS1 regulate KDM5B expression, and KDM5B potentially contributed to atherosclerosis by regulating endothelial inflammation.

Using a scRNA‐seq analysis of the carotid artery exposed to proatherogenic d‐flow, we profiled the endothelial expression of histone lysine demethylases. D‐flow responsive or unresponsive gene expression patterns were observed among the 19 KDM family members. Remarkably, KDM5B was upregulated in the pro‐atherogenic d‐flow stimulated endothelial subpopulations but not in the endothelial cells under healthy stable flow conditions. KDM5B expression is unique to the KDM5 subfamily, as there was no change in the expression of other KDM5 subfamily members in response to disturbed flow. In addition to KDM5B, KDM7A and KDM7C, members of the KDM7 subfamily, were upregulated (Figure S1). The KDM7 subfamily, which belongs to the Jmj‐KDM family, is an emerging class of transcriptional coactivators because its members erase the repressive marks H3K9me2/1, H3K27me2/1 and H4K20me1 [53]. KDM7A demethylates H3K9me2 and H3K27me3, which is required for NF‐κB activation and inflammatory gene expression, in turn promoting leukocyte adhesion and exacerbating inflammation in mice [54, 55]. It increases ICAM‐1 expression level induced by TNF‐α and enhances its stability through the lysosomal pathway in the brain [56]. As KDM7A responds to d‐flow and is involved in inflammation, it is worth examining whether it participates in the development of atherosclerosis. KDM7C (also called PHD Finger Protein 2, PHF2), as a protein code and transcription regulatory gene, is a member of the Jumonji‐C domain (JmjC) and contains a zinc finger‐like PHD (plant homeodomain) [57, 58]. It mediates the demethylation of H3K9me2, subsequently activating the expression of target genes [59, 60]. Recent studies have shown that KDM7C regulates interleukin 4 production in CD4^+^ T cells and may play a role in the development of immune diseases [61] and that it directly destabilises SREBP1c and reduces SREBP1c‐dependent lipogenesis [62]. To date, there have been no reports on the role of KDM7C in atherosclerosis.

Endothelial cells transduce flow signals into intracellular changes through mechanosensing and mechanosignal transduction by recognising fluid shear stress through mechanosensors, including plasma membrane proteins (e.g., the cation channels Piezo1 and P2X4, Notch, protein kinases, GPCRs, plexin D1 and integrins), membrane‐associated structures (e.g., caveolae, the glycocalyx and primary cilia) and cell–cell junctional molecules (e.g., PECAM1, VE‐cadherin and VEGFR2) [63]. By analysing our scRNA‐seq datasets from the LCA after PCL [42] for the candidate mechanosensors that are differentially expressed in sh*Kdm5b* HUVECs, we showed that the known mechanosensors *Piezo1*, *Pdgfb* and *Notch4* were upregulated mainly in the d‐flow‐associated EC subclusters, but not in the endothelial cells under healthy stable flow conditions, indicating their potential role in regulating KDM5B expression. However, the other candidate mechanosensors, *Sdc4* and *Cav1*, were not induced by d‐flow in endothelial cells (Figure S4). Piezo1 is known to mediate either atheroprotective or pro‐atherogenic flow‐dependent endothelial cell responses [63]. For example, Piezo1 senses stable flow to promote the release of NO from the endothelium, thereby affecting the tension of local blood vessels and playing an anti‐atherosclerosis role [64], while disturbed flow leads to Piezo1‐mediated integrin activation, resulting in focal adhesion kinase‐dependent NF‐κB activation [65]. Although epigenetic pathways respond to temporal and spatial variations in flow and pressure, particularly hemodynamically disturbed blood flow [66], whether Piezo1 participates in KDM5B expression is unknown. In this study, we showed that Piezo1 deficiency downregulated KDM5B expression and that Piezo1 activation by Yoda1 in endothelial cells upregulated KDM5B. Downstream of Piezo1, we found that the transcription factors ETS1 and c‐JUN were likely responsible for KDM5B expression. However, further investigation is required to fully understand the mechanotransduction pathway downstream of Piezo1 in d‐flow induced KDM5B expression. In addition, we cannot exclude the involvement of other mechanosensors (e.g., *Notch4*) in mediating d‐flow induced KDM5B expression.

Although the histone methylation level is associated with transcriptional inhibition and gene expression, histone methylation inhibitors have not been extensively studied compared to other epigenetic inhibitors. KDM5B functions as a histone demethylase and has been a hot drug target in recent years. Several studies have shown that KDM5B is linked to cancer immunotherapy and that its inhibitor AS‐8351 can suppress EwS cell proliferation and inhibit tumour growth in nude mouse models [67, 68, 69]. Another small molecule inhibitor of KDM5B, the inhibitor GS‐5801, was found to inhibit HBV viral activity in a model of primary human hepatocyte infection [70]. Moreover, GSK467 was identified as a potent and selective inhibitor of KDM5B and was reported to constrain the transition of cardiac fibroblasts to profibrogenic myofibroblasts and ameliorate cardiac fibrosis [33, 71]. A recent study revealed that the KDM5B inhibitor TK‐129 can block KDM5B‐related Wnt pathway activation and may represent a novel KDM5‐targeting lead compound for cardiac remodelling and fibrosis [72]. In our study, we found that KDM5‐IN‐1, an inhibitor of KDM5B, could also increase H3K4me3 levels in endothelial cells induced by disturbed flow. Whether these inhibitors contribute to the formation of atherosclerotic plaques is still unclear, which is a promising direction for further investigation.

In summary, we identified that the mechanosensor Pleizo1 regulates KDM5B expression and H3K4me3 level in the disturbed flow stimulated endothelial cells. Piezo1 senses d‐flow and upregulates KDM5B expression through ETS1 and c‐JUN at the transcriptional level in vascular endothelial cells. Functionally, KDM5B deletion reduced endothelial inflammation and attenuated d‐flow induced carotid atherosclerotic plaque formation. Our findings may lead to potential novel approaches for the prevention and treatment of atherosclerosis.

## Author Contributions

**Lili Wu:** data curation (equal), funding acquisition (equal), methodology (equal), project administration (equal), resources (equal), writing – original draft (lead), writing – review and editing (equal). **Shanshan Jiang:** writing – original draft (equal). **Xiao Zhou:** methodology (equal). **Wei Li:** data curation (equal). **Jiaqi Ke:** methodology (equal). **Ziting Liu:** validation (equal). **Lijie Ren:** funding acquisition (equal). **Qiongyu Lu:** methodology (equal). **Fengchan Li:** formal analysis (equal), software (equal). **Chaojun Tang:** project administration (equal), writing – original draft (equal), writing – review and editing (equal). **Li Zhu:** writing – original draft (supporting), writing – review and editing (supporting).

## Conflicts of Interest

The authors declare no conflicts of interest.

## Supporting information


Appendix S1.

## Data Availability

The sc‐RNA sequencing data that support the findings of this study have been deposited in the SRA under the accession code PRJNA722117.
