# Supplementary material for: Endothelial KDM5B Regulated by Piezo1 Contributes to Disturbed Flow Induced Atherosclerotic Plaque Formation
Source: J Cell Mol Med. 2024 Dec 6;28(23):e70237. doi: 10.1111/jcmm.70237 (PMC11624123; doi:10.1111/jcmm.70237)
Supplement: Supplementary file 1 — Appendix S1. [file JCMM-28-e70237-s001.docx]

**Supplementary Information**

**
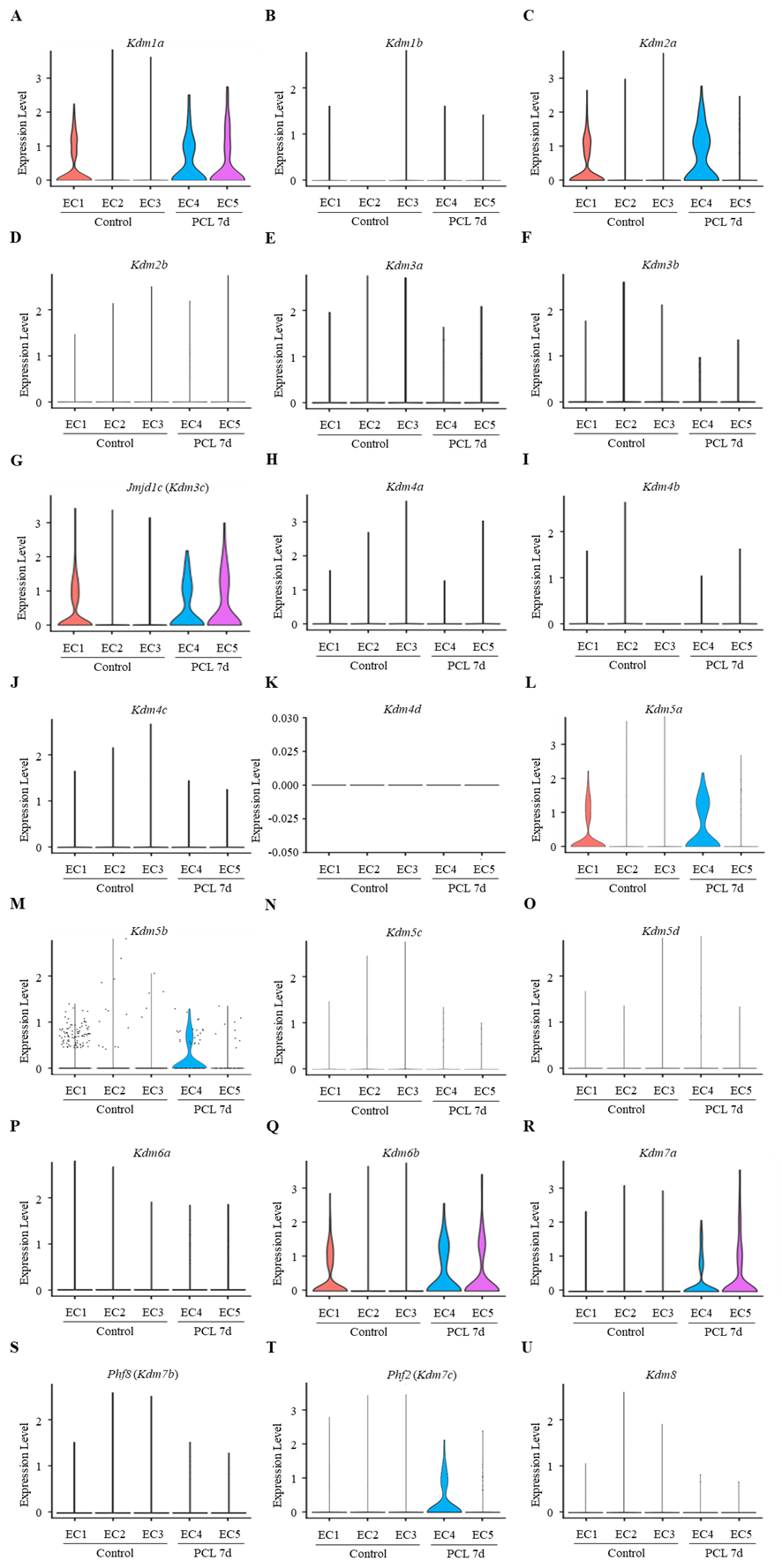
**

**Figure S1. Single-cell transcriptional profiling reveals expression of histone demethylases in vascular endothelial cell clusters**. (A-B) Violin plots of *Kdm1a* and *Kdm1b* expression in five EC clusters. (C-D) Violin plots of *Kdm2a* and *Kdm2b* expression in five EC clusters. (E-G) Violin plots of *Kdm3a*, *Kdm3b*, and *Jmjd1c* (*Kdm3c*) expression in five EC clusters. (H-K) Violin plots of *Kdm4a*, *Kdm4b*, *Kdm4c*, and *Kdm4d* expression in five EC clusters, except for *Kdm4f* and *Kdm4e*, which were not found in our scRNA-seq dataset. (L-O) Violin plots of *Kdm5a*, *Kdm5b*, *Kdm5c*, and *Kdm5d* expression in five EC clusters. (P-Q) Violin plots of *Kdm6a* and *Kdm6b* expression in five EC clusters. (R-T) Violin plots of *Kdm7a*, *Phf8* (*Kdm7b*), and *Phf2* (*Kdm7c*) expression in five EC clusters. (U) Violin plots of *Kdm8* expression in five EC clusters.


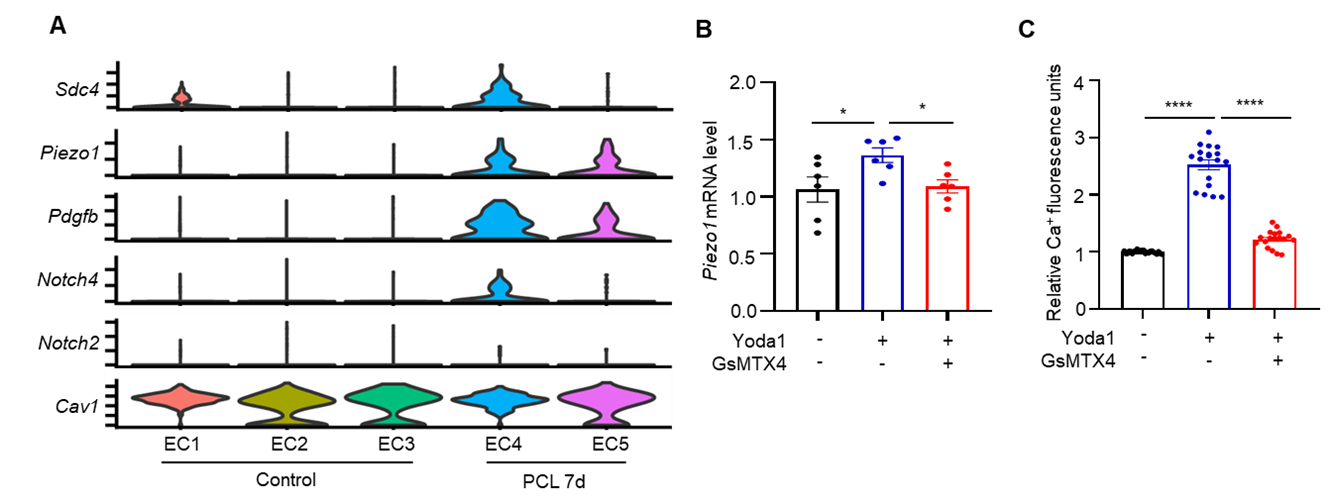


**Figure S2.** **Piezo1 is a mechanosensory protein and promotes calcium influx.** (A) The expression of mechanosensors that were differentially expressed in the sh*Kdm5b* HUVECs RNA-Seq database was analyzed by single-cell sequencing. Violin plots showing *Sdc4*, *Piezo1*, *Pdgfb*, *Notch4*, *Notch2* and *Cav1* expression in control and PCL-stimulated ECs. (B & C) Yoda1 promotes Piezo1 expression and activation, while GsMTX4 reverses this phenomenon. **P* < 0.05, *****P* < 0.0001 by unpaired Student’s *t*-test.


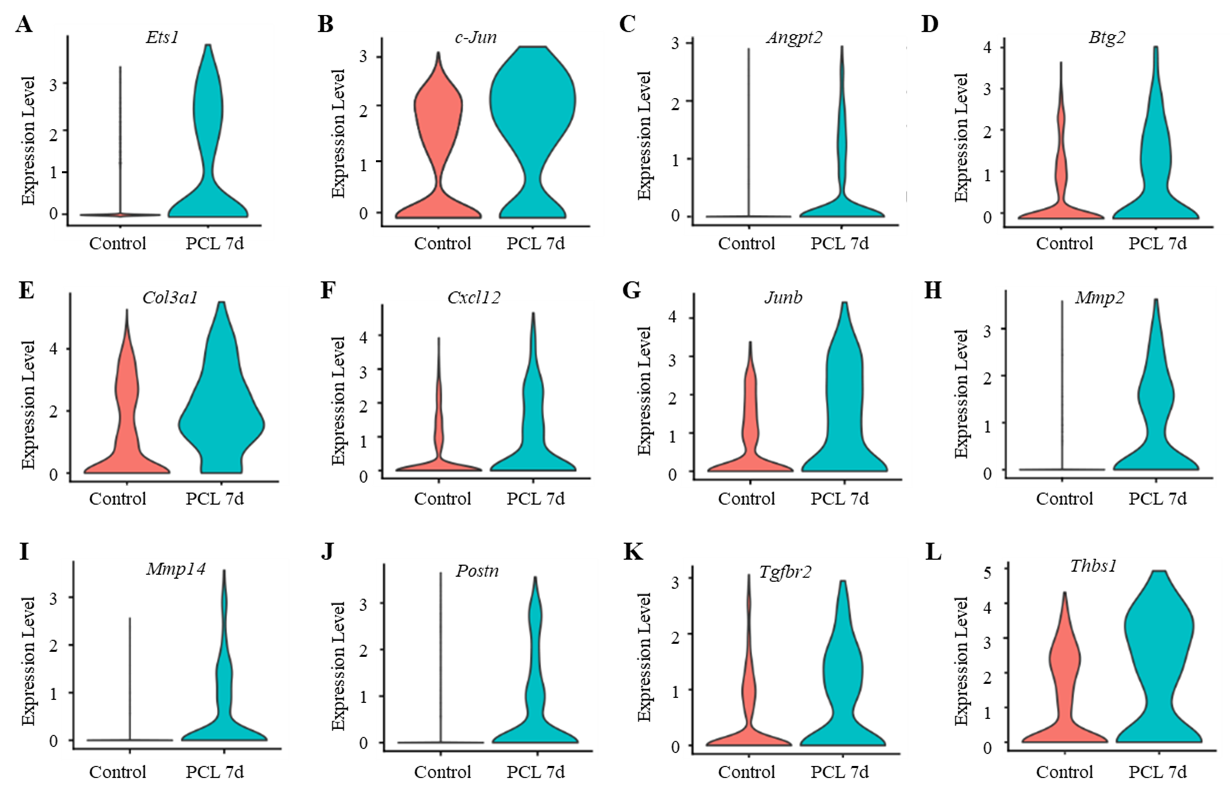


**Figure S3.** **Disturbed blood flow upregulates gene expression in response to mechanical stimuli in endothelial cells**. Violin plots showing *Ets1* (A), *c-Jun* (B), *Angpt2* (C), *Btg2* (D), *Col3a1* (E), *Cxcl12* (F), *Junb* (G), *Mmp2* (H), *Mmp14* (I), *Postn* (J), *Tgfbr2* (K), and *Thbs1* (L) expression in Control and PCL7d stimulated ECs.

**
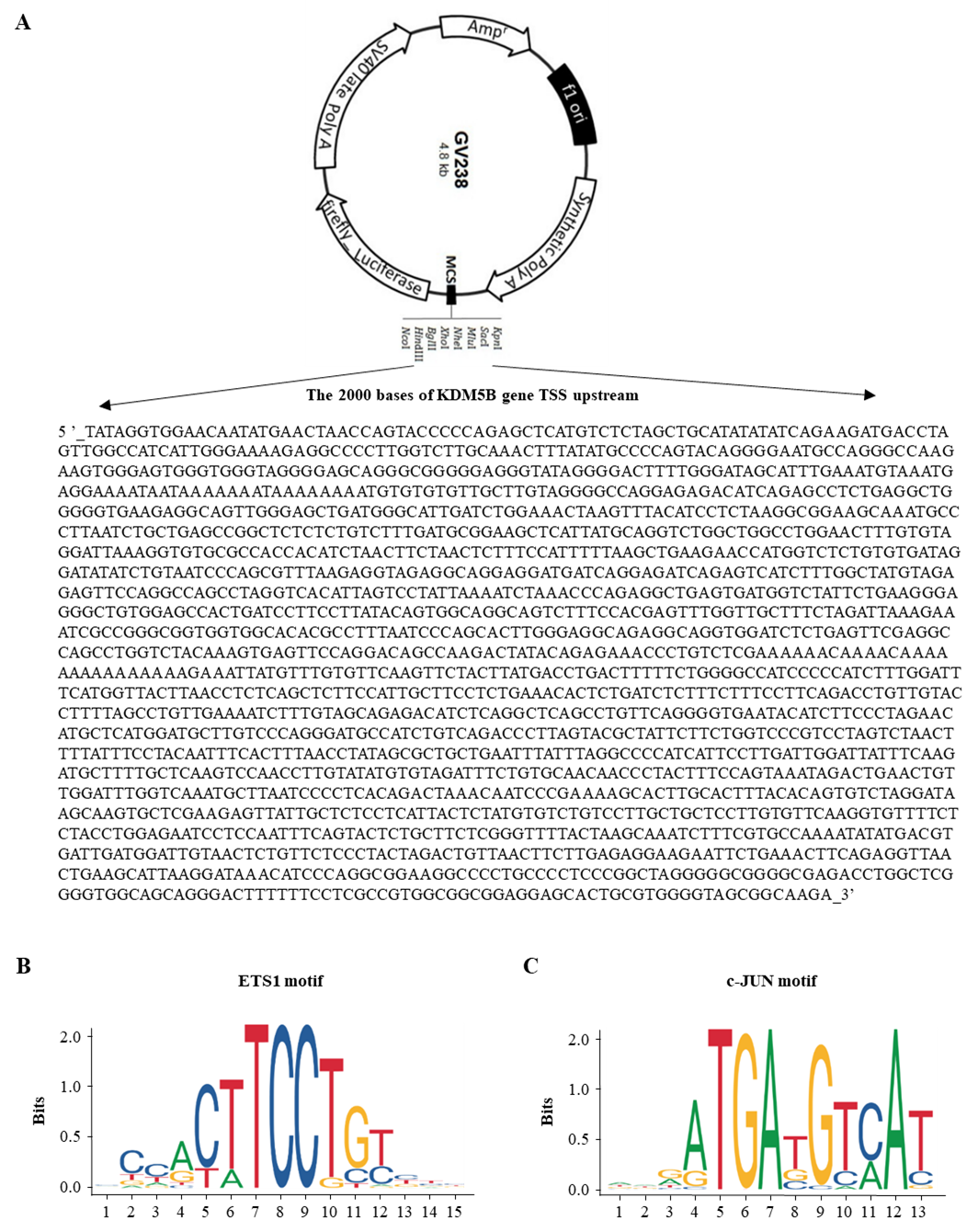
Figure S4. Schematic graph of the constructed KDM5B promoter region constructed with the firefly luciferase reporter gene plasmid.** (A) The 2000 bases of the KDM5B gene upstream of the transcription start site. (B) The ETS1 binding motif from the JASPAR database. (C) The c-JUN binding motif from the JASPAR database.


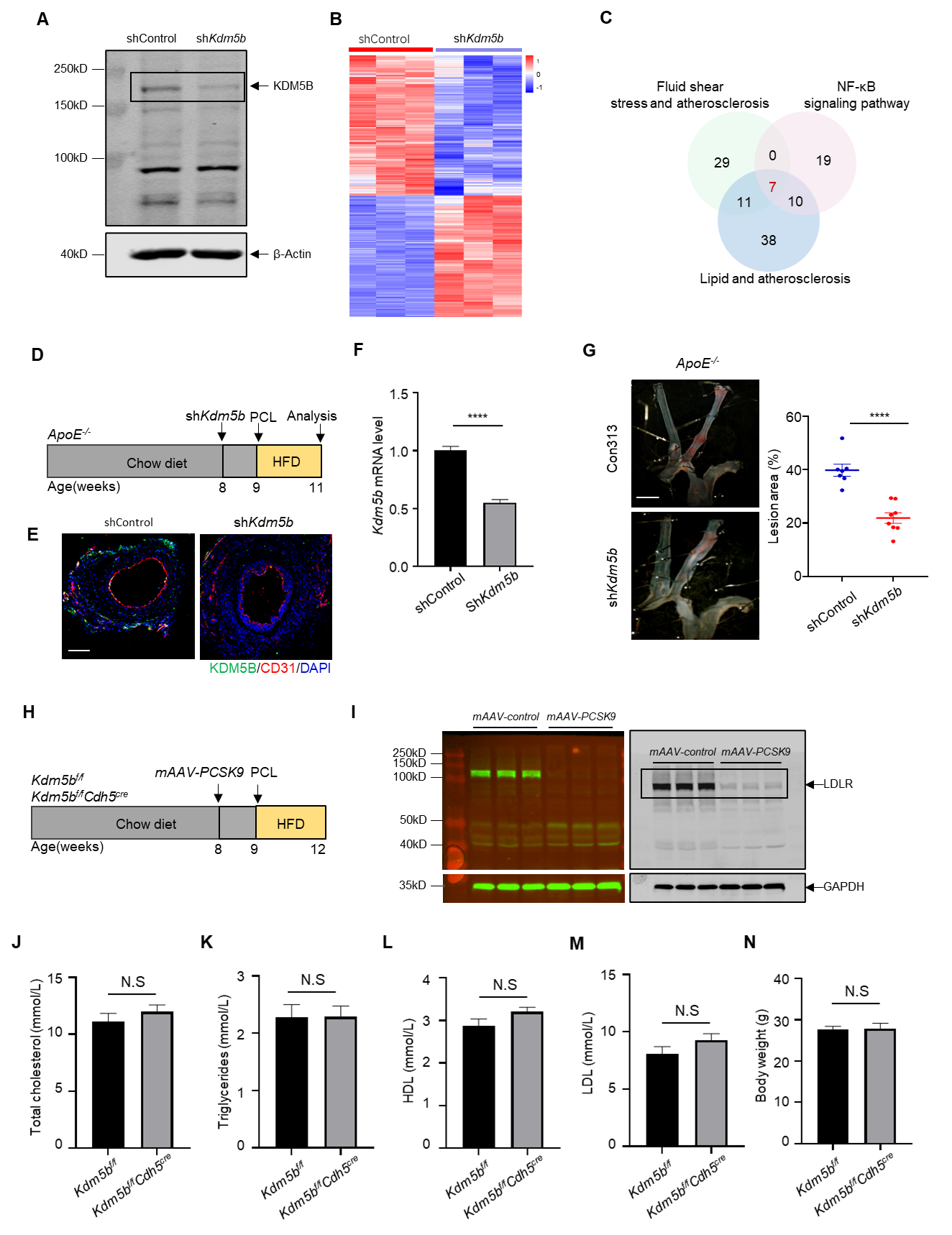


**Figure S5. RNA-seq of KDM5B knockdown HUVECs and endothelial KDM5B deficiency had no effect on lipid profiles or body weight.** (A) KDM5B protein expression in lentivirus-mediated Con313 and sh*Kdm5b* HUVECs was analyzed by Western blotting and normalized to β-actin. (B) Heatmap was used to show the differentially expressed genes in Con313 and sh*Kdm5b* HUVECs. (C) Venn diagram showing the genes involved in these signaling pathways. (D) Schematic representation of lentiviral-mediated carotid KDM5B knockdown and atherosclerotic plaque formation. (E) Immunostaining was used to determine KDM5B (green) expression in sh*Kdm5b*-infected carotid arteries and control Con313-infected arteries. Bar = 100 μm. (F) RT-qPCR was used to detect the knockdown efficiency of *Kdm5b* in sh*Kdm5b* lentivirus infected carotid arteries and control Con313 infected arteries. (n=4 per group). *****P* < 0.0001 by unpaired Student’s *t*-test. (G) *ApoE^-/-^* mice were subjected to PCL with shControl or KDM5B shRNA instillation. After 2 weeks on a HFD, lipid deposition (in red) on the mouse LCA was analyzed by *en face* Sudan IV staining. The lesion surface area was quantified and is displayed as the percentage area of the LCA. Bar = 1 mm. (n ≥ 7 mice per group). The data are presented as the mean ± SEM. *****P* < 0.0001 according to unpaired Student’s *t*-test. (H) *Kdm5b^f/f^* mice (n = 12) and *Kdm5b^f/f^ Cdh5^cre^* mice (n = 8) were injected with *mAAV-PCSK9* (5 × 10^11^ VG/mouse). After one week, the mice were subjected to PCL and fed a HFD for 3 weeks. (I) Western blot analysis showing the expression level of LDL receptors in liver tissue samples obtained from animals injected with *mAAV-PCSK9* or *mAAV*-*control*. Left image is the color picture. (J-N) No overt differences in plasma cholesterol (J), triglyceride (K), HDL (L), or LDL (M) levels or body weight (N) were detected between *Kdm5b^f/f^ Cdh5^cre^* mice and *Kdm5b^f/f^* mice infected with *mAAV*-*PCSK9*^D377Y^. n ≥ 8 mice per group. N.S, not significant, by unpaired Student’s *t*-test.

**Supplementary Tables S1.** Primers used for RT-qPCR

| **Name** | **Forward (5’--3’)** | **Reverse (5’--3’)** |
| --- | --- | --- |
| *Kdm5a* (mouse) | TGCAAATGAGACAACGGAAAGG | CTGTCATCGCACCCATCACA |
| *Kdm5b* (mouse) | CTGTGGGCTCACATATCAGGG | TGGGGTTTGTACTCCTTGTCC |
| *Kdm5c* (mouse) | GACCCATCGCCGAGAAGTC | TCGGGGAGTAAACCTGAAGTT |
| *Kdm5d* (mouse) | ATGAAGCCAGGATCTGACGAC | GCCAAGAGGATCGCGGAAT |
| *Piezo1* (mouse) | CCTGTTACGCTTCAATGCTCT | GTGTAGGCATATCTGAAAGGCAA |
| *Gapdh* (mouse) | AGGTCGGTGTGAACGGATTTG | GGGGTCGTTGATGGCAACA |
| *NFkb1* (human) | AACAGAGAGGATTTCGTTTCCG | TTTGACCTGAGGGTAAGACTTCT |
| *Rela* (human) | GTGGGGACTACGACCTGAATG | GGGGCACGATTGTCAAAGATG |
| *Bcl2* (human) | GGTGGGGTCATGTGTGTGG | CGGTTCAGGTACTCAGTCATCC |
| *Vcam1* (human) | TTTGACAGGCTGGAGATAGACT | TCAATGTGTAATTTAGCTCGGCA |
| *Icma1* (human) | ATGCCCAGACATCTGTGTCC | GGGGTCTCTATGCCCAACAA |
| *Kdm5b* (human) | AGTGGGCTCACATATCAGAGG | CAAACACCTTAGGCTGTCTCC |
| *Gapdh* (human) | GGAGCGAGATCCCTCCAAAAT | GGCTGTTGTCATACTTCTCATGG |
